# ^19^F NMR‐based solvent accessibility profiling reveals tryptophan ring‐flip dynamics in a protein

**DOI:** 10.1002/pro.70307

**Published:** 2025-09-13

**Authors:** Soichiro Kawagoe, Hiroyuki Kumeta, Tomohide Saio

**Affiliations:** ^1^ Institute of Advanced Medical Sciences Tokushima University Tokushima Japan; ^2^ Faculty of Advanced Life Science Hokkaido University Sapporo Hokkaido Japan; ^3^ Fujii Memorial Institute of Medical Sciences, Institute of Advanced Medical Sciences Tokushima University Tokushima Japan

**Keywords:** ^19^F NMR, aromatic ring flipping, CPMG relaxation dispersion, protein breathing motion, solvent PRE

## Abstract

Aromatic ring flipping is a key component of protein conformational dynamics, particularly in facilitating breathing motions that involve cooperative rearrangements of hydrophobic cores. While phenylalanine and tyrosine flipping have been extensively studied, tryptophan flipping has often been overlooked due to assumptions about limited mobility arising from its bulkiness and the asymmetric structure of the indole ring. Here, we report a ^19^F NMR–based approach to probe the dynamic flipping of Trp23 in the DNA‐binding domain (DBD) of heat shock factor 1 (Hsf1). We incorporated 5‐fluorotryptophan into the Hsf1 DBD and monitored Trp23 flipping in solution using ^19^F NMR. Temperature‐dependent analysis corroborated with relaxation dispersion experiments revealed interconversion between two conformers of Trp23, which correspond to the flip‐in and flip‐out states observed in crystal structures. Furthermore, D_2_O isotope shifts and solvent paramagnetic relaxation enhancement experiments revealed population shifts between the solvent‐exposed flip‐out state and the buried flip‐in ground state. These results establish that Trp23 undergoes flipping between buried and solvent‐exposed conformations in solution. This study represents the first application of ^19^F NMR‐based solvent accessibility profiling to characterize aromatic ring flipping. The strategy presented here is broadly applicable to studies of aromatic dynamics in diverse protein systems.

**Abbreviations**
NMR: nuclear magnetic resonance
Hsf1: heat shock factor 1
DBD: DNA binding domain
CSP: chemical shift perturbation
sPRE: solvent paramagnetic relaxation enhancement
5F‐Trp: 5‐fluorotryptophan
*R*_1_
: longitudinal relaxation rates
CPMG RD: Carr‐Purcell‐Meiboom‐Gill relaxation dispersion

## 1 INTRODUCTION

Protein dynamics are fundamental to biological function, mediating processes ranging from molecular recognition to allosteric regulation (Eisenmesser et al., 2002). Among the various contributors to these dynamics, the conformational dynamics of aromatic amino acids—phenylalanine, tyrosine, histidine, and tryptophan—play a particularly important role. Aromatic residues typically occupy a substantial portion of the hydrophobic core and are frequently organized into specific pairwise or clustered interactions (Burley & Petsko, 1985; Wagner et al., 1976; Wüthrich & Wagner, 1975). Despite their buried location, aromatic side chains are not static; rather, they undergo dynamic flipping. This flipping requires cooperative rearrangement of surrounding residues, often referred to as protein breathing motions (Akke & Weininger, 2023; Dreydoppel et al., 2025; Hattori et al., 2004; Kasinath et al., 2015; Kulkarni & Söderhjelm, 2023; Mariño Pérez et al., 2022; Skalicky et al., 2001; Weininger et al., 2013; Weininger et al., 2014). Intriguingly, aromatic ring flipping has also been shown to respond to protein–ligand interactions, suggesting its relevance not only to conformational plasticity but also to molecular recognition (Yang et al., 2015). Consequently, the ability to control or harness ring flipping has attracted interest in protein engineering and design.

Studies of ring flipping have primarily focused on phenylalanine and tyrosine, which are symmetric and relatively compact. In contrast, tryptophan flipping has remained understudied due to assumptions of limited mobility arising from the bulkiness and asymmetry of the indole ring (Akke & Weininger, 2023; Dreydoppel et al., 2021; Hansson et al., 1998; Kinsey et al., 1981). However, recent studies highlight the functional importance of tryptophan dynamics in proteins. For example, crystallographic analyses of the NS2B‐NS3 protease have shown that tryptophan residues can adopt alternative rotameric states upon ligand binding (Phoo et al., 2020). An NMR study using site‐specifically incorporated fluorotryptophan into NS2B‐NS3 detected two distinct signals corresponding to rotameric states in the ligand‐free state, which shifted to a single peak upon ligand binding in slow exchange on the NMR timecale (Qianzhu et al., 2024). Such studies demonstrate the potential of ^19^F NMR as a powerful tool for probing tryptophan ring flipping with its high sensitivity, wide chemical shift range, and ability to site‐specifically monitor fluorinated side chains without background signals (Gronenborn, 2022). Moreover, recent developments have further expanded the application of ^19^F NMR to characterize the kinetics of tyrosine ring flipping in a protein (Lu et al., 2024). Nevertheless, NMR signals from aromatic residues do not always appear as well‐resolved peaks for individual rotameric states (Mariño Pérez et al., 2022), and a robust ^19^F NMR‐based strategy for characterizing the dynamics of tryptophan ring flipping in solution—including its kinetics, energetics, and structural features—remains to be established.

Here, we present an integrative ^19^F NMR‐based approach to monitor tryptophan ring‐flip dynamics in solution by combining temperature‐dependent analysis of chemical shift perturbation (CSP), Carr‐Purcell‐Meiboom‐Gill relaxation dispersion (CPMG RD), D₂O isotope shifts, and solvent paramagnetic relaxation enhancement (sPRE). Using the DNA‐binding domain (DBD) of human heat shock factor 1 (Hsf1) as a model, we focused on Trp23, which adopts two distinct conformations in crystal structures and is predicted to exhibit flip state‐dependent solvent exposure. Our results suggest a generalizable framework for profiling the dynamics of aromatic ring flips using ^19^F NMR.

## 2 RESULTS

### 2.1 Structural evidence of two‐state flipping equilibrium of Trp23 in the Hsf1 DBD

**FIGURE 1:**
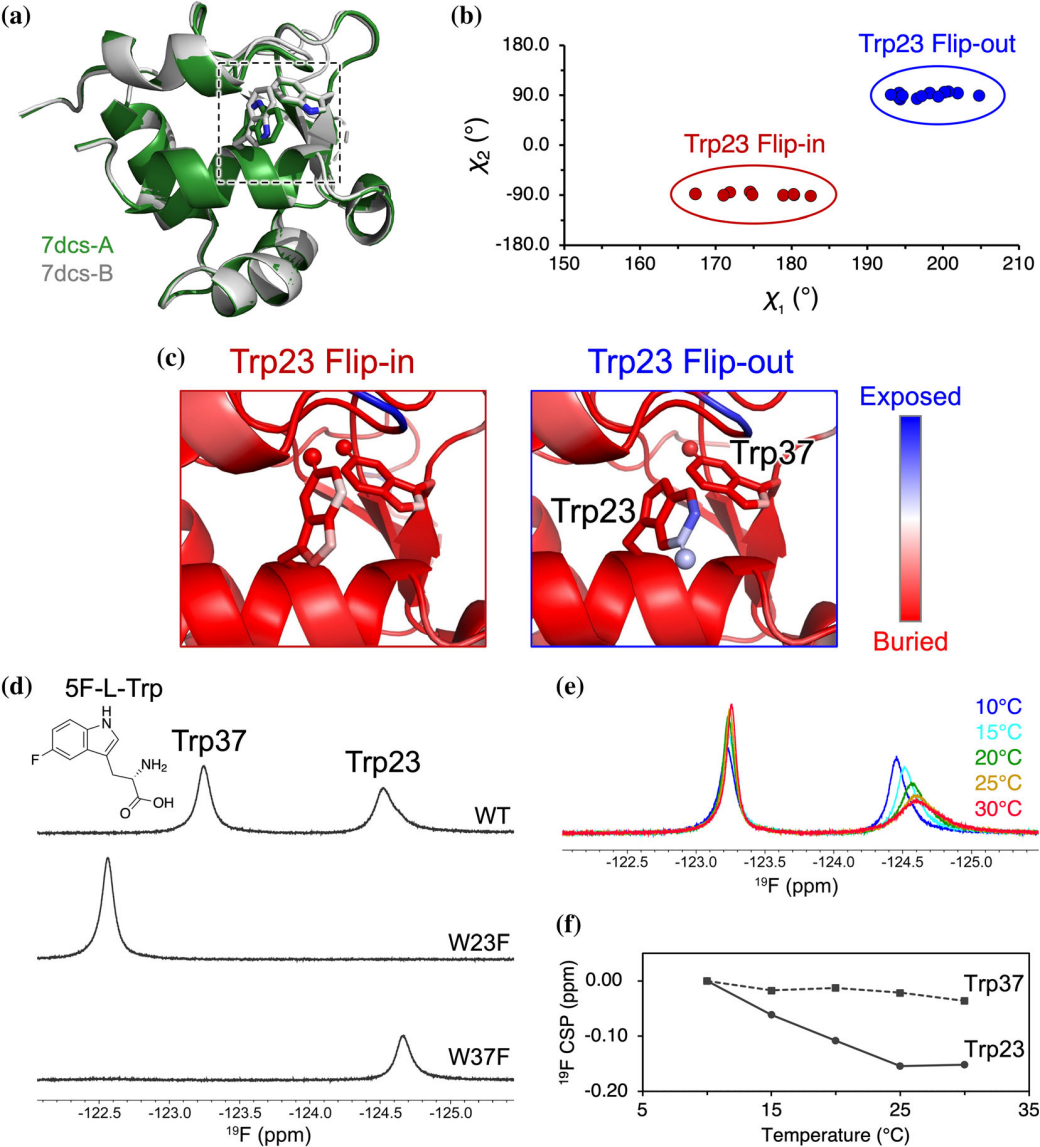
Flip‐in and flip‐out conformations of Trp23 in the Hsf1 DBD. (a) Crystal structures of the Hsf1 DBD from PDB entry 7dcs, shown as a cartoon representation with chains A (green) and B (gray). Trp23 and Trp37 are highlighted as sticks. (b) χ₁ − χ_2_ plots of Trp23 analyzed from PDB structures, corresponding to the data presented in Table S1. (c) Enlarged views of Trp23 in the “flip‐in” (buried) and “flip‐out” (solvent‐exposed) conformations. The local surface exposure was mapped onto the protein structure based on the solvent‐accessible surface area (SASA) calculated using PyMOL, colored from blue (exposed) to red (buried). (d) ^19^F NMR spectra of the DBD wild‐type (WT), W23F, and W37F mutants, showing the distinct signals originating from site‐specifically incorporated 5‐fluorotryptophan. All ^19^F spectra were recorded at 20°C. (e) Temperature‐dependent ^19^F NMR spectra of the DBD WT, indicating the dynamic equilibrium between Trp23 conformers. (f) Plot showing the CSP of the ^19^F resonance peaks as a function of temperature.

The DBD of human Hsf1 contains two tryptophan residues, Trp23 and Trp37 (Kawagoe et al., 2022). Multiple crystal structures of the DBD in both apo and DNA‐bound forms have been reported (Feng et al., 2021; Neudegger et al., 2016). Trp23 exhibits structural polymorphism between two rotameric states in the DNA‐bound forms, whereas the backbone conformation and the side chain of the buried Trp37 are highly conserved (Figures 1a–c and S1 and Table S1). In one conformation of Trp23, the indole NH group is buried, and the C5 and C6 positions of the benzene ring point outward and are solvent‐exposed—defined here as the “flip‐out” state. In the alternative “flip‐in” state, the benzene ring faces inward and is more buried (Figure 1c). This flipping of the indole ring involves not only a 180° rotation around the χ_2_ dihedral angle but also a conformational change in χ_1_, representing a cooperative conformational transition involving both χ_1_ and χ_2_ dihedral angles (Figure 1b). Here we use the term “ring flipping” to describe this exchange process that includes cooperative conformational change of the Trp23 indole ring. Similarly, multiple Trp conformations are seen for Trp15 in the DBD of Hsf2, a homolog of Hsf1 (Figure S1). The high sequence conservation of Trp23 across the Hsf family (Figure S2) further supports the idea that this conformational duality may represent a conserved biophysical feature of Hsf family DBDs. However, the possibility remains that the observed conformations are influenced by crystal packing, and whether Trp23 undergoes dynamic flipping in solution has not been fully investigated.

We exploited ^19^F NMR and monitored the conformational states of Trp23 in solution. Tryptophan residues of the Hsf1 DBD with 5‐fluorotryptophan (5F‐Trp), in which the proton at the C5 position was substituted with a fluorine atom, enabled site‐specific detection of the indole conformation by ^19^F NMR. In the 5F‐Trp labeled DBD wild‐type (WT), two ^19^F signals were observed (Figure 1d). These were assigned to Trp23 and Trp37 based on spectra of W37F and W23F mutants, respectively (Figure 1d). Notably, the chemical shift of the Trp37 signal differed by approximately 0.7 ppm between the WT and the W23F mutant. This shift likely reflects not only structural changes caused by the mutation but also the close spatial proximity between Trp23 and Trp37 in the WT protein due to aromatic ring current, which may cause local magnetic effects (Gomes & Mallion, 2001). Indeed, aromatic residues are known to form clusters through specific π–π interactions within protein hydrophobic cores, leading to characteristic shielding or deshielding effects (Burley & Petsko, 1985; Burley & Petsko, 1989).

We then performed ^19^F NMR measurements at varying temperatures and examined whether the Trp23 signal reflects a static conformation or conformational exchange. While the Trp37 signal exhibited only minimal changes in chemical shift and linewidth, the Trp23 signal progressively became broader and shifted upfield with increasing temperature (Figures 1e and S3), suggesting intermediate exchange behavior (Bain, 2003). Taken together with the crystal structure observations showing flip‐in/out conformations of Trp23, these results suggested that Trp23 undergoes temperature‐dependent conformational exchange between the flip‐in and flip‐out states.

### 2.2 ^19^F CPMG RD experiments for probing Trp23 flipping

**FIGURE 2:**
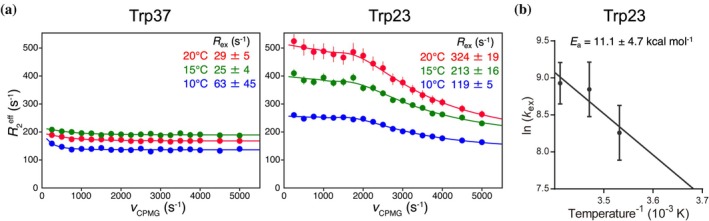
^19^F CPMG RD experiments for probing tryptophan dynamics. (a) ^19^F CPMG RD profiles of Trp37 and Trp23, recorded at 800 MHz proton frequency at 10°C, 15°C, and 20°C. (b) The calculated exchange rates (*k*_ex_) of Trp23 are plotted following the Arrhenius equation at different temperatures.

To further analyze the Trp23 flipping motion, we exploited ^19^F CPMG RD experiments (Krempl & Sprangers, 2023; Overbeck et al., 2020). ^19^F CPMG RD profiles were recorded at three different temperatures: 10°C, 15°C, and 20°C. Consistent with the CSP results, a larger dispersion was observed for Trp23 compared to Trp37, indicating chemical exchange (Figure 2a). A global fit according to a two‐site exchange model gives exchange rates *k*_ex_ of 3860 ± 1430 s^−1^ at 10°C, 6940 ± 2560 s^−1^ at 15°C, and 7540 ± 2120 s^−1^ at 20°C (Table 1). This temperature dependence derived the activation energy (*E*_a_) of 11.1 ± 4.7 kcal mol^−1^ (Figure 2b and Tables 1 and S4). This exchange rate, on the order of thousands s^−1^, is typical for the ring‐flip dynamics of aromatic amino acid residues buried in protein cores (Akke & Weininger, 2023; Dreydoppel et al., 2025; Kulkarni & Söderhjelm, 2023; Mariño Pérez et al., 2022; Skalicky et al., 2001; Weininger et al., 2014). Moreover, the observed population bias and *E*_a_ (Table 1) coincide with the typical interaction energy of several hydrogen bonds or π–π stacking (Burley & Petsko, 1985; Gallivan & Dougherty, 2000; McGaughey et al., 1998; Singh et al., 2009), suggesting the exchange involves concerted rearrangement of interaction mode around Trp23. Thus, the chemical exchange profile obtained from the ^19^F CPMG RD experiments strongly supports that it reflects the flipping motion of Trp23.

**TABLE 1:** Kinetic and energetic parameters of Trp23 calculated from ^19^F CPMG experiments.

Temp (°C)	*k*_ex_ (s^−1^)	*E*_a_ (kcal mol^−1^)	*p*_major_ (%)	*p*_minor_ (%)	*K*_eq_	Δ*G*° (kcal mol^−1^)	Δ*H*° (kcal mol^−1^)	Δ*S*° (cal mol^−1^ K^−1^)
10	3860 ± 1430	11.1 ± 4.7	96.5 ± 1.8	3.5	0.0361 ± 0.0194	1.87 ± 0.30	7.63 ± 2.76	19.0 ± 9.6
15	6940 ± 2560	11.1 ± 4.7	96.2 ± 2.1	3.8	0.0391 ± 0.0221	1.82 ± 0.32	7.63 ± 2.76	19.0 ± 9.6
20	7540 ± 2120	11.1 ± 4.7	94.7 ± 1.8	5.3	0.0561 ± 0.0204	1.62 ± 0.20	7.63 ± 2.76	19.0 ± 9.6

### 2.3 D_2_O‐induced ^19^F isotope shifts suggested the presence of the flip‐out state in solution

**FIGURE 3:**
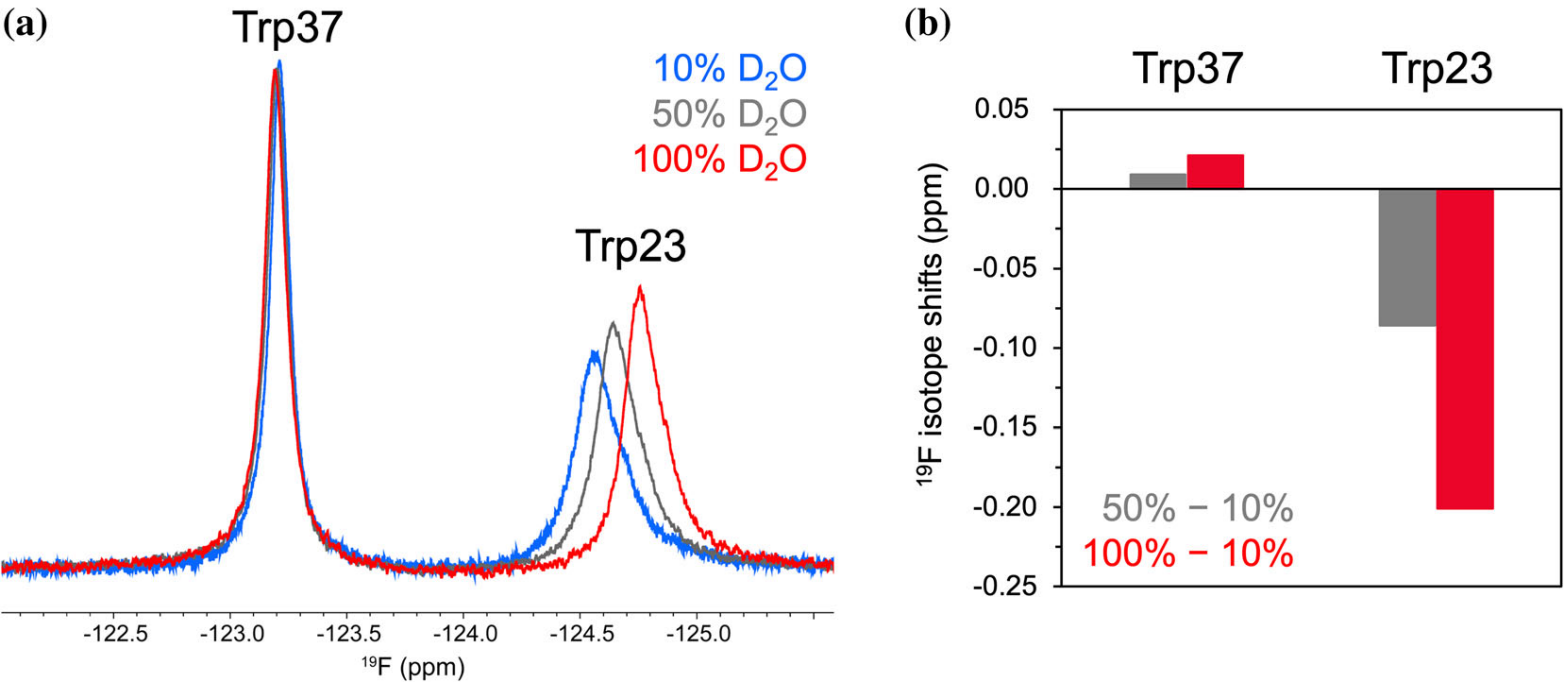
^19^F D_2_O‐induced isotope shifts of the Hsf1 DBD. (a) Overlay of the three ^19^F spectra of Hsf1 DBD in 10% (blue), 50% (gray), and 100% (red) D_2_O, respectively. (b) ^19^F isotope shifts relative to the 10% D_2_O condition are plotted as bar graphs, with gray and red indicating 50 % to 10% and 100 % to 10% comparisons, respectively.

The flipping state of Trp23 was assessed in terms of solvent exposure. We measured ^19^F chemical shift differences in 10%, 50%, and 100% D_2_O for the Hsf1 DBD, as D_2_O‐induced isotope shifts reflect the solvent accessibility of the fluorine atom in proteins (Kitevski‐LeBlanc et al., 2009; Osten et al., 1985; Zhu et al., 2025). The ^19^F signal from Trp37 remained essentially unchanged across D_2_O concentrations, consistent with its buried environment and limited hydration (Figures 1a,c and 3a,b). In contrast, the Trp23 signal shifted upfield in a D_2_O concentration–dependent manner, showing a total shift of approximately 0.2 ppm between 10% and 100% D_2_O (Figure 3a,b). The observed isotope shift is comparable in magnitude to values reported for polypeptide hormones labeled with 4F‐Phe (Hou et al., 2017), indicating that the C5 position of Trp23 resides in a relatively solvent‐exposed environment. These results are consistent with the flip‐out conformation observed in crystal structures, in which the fluorinated benzene ring is oriented toward the solvent.

### 2.4 Tracking the population shifts of Trp23 flipping states by ^19^F sPRE

**FIGURE 4:**
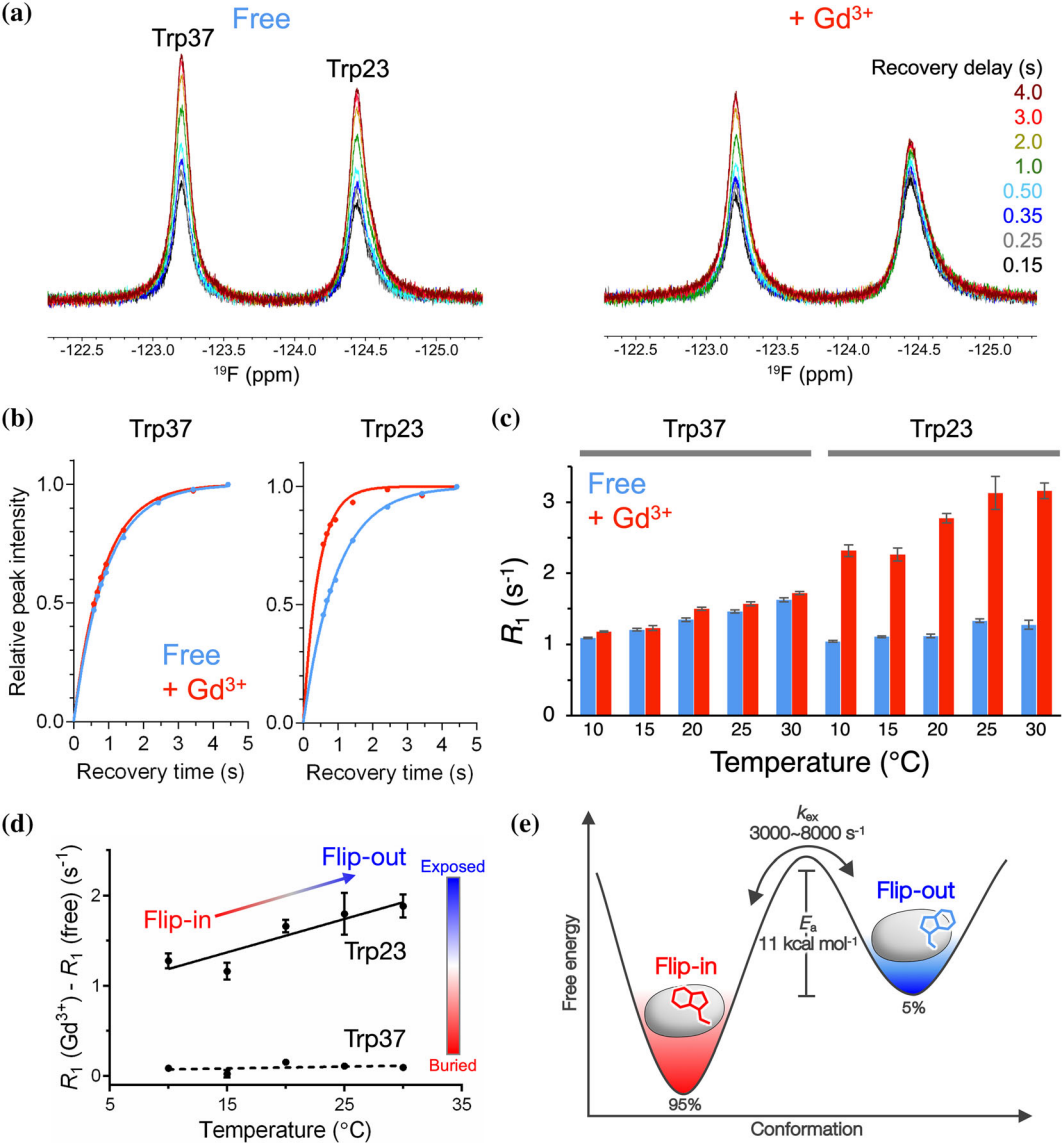
Temperature‐dependent ^19^F sPRE of the Hsf1 DBD. (a) Overlay of ^19^F NMR spectra of Hsf1 DBD recorded with varying recovery delays (d_1_ = 0.15 to 4.0 s) in the absence (Free) and presence (+Gd^3+^) of 5 mM gadodiamide. (b) Measurements of ^19^F *R*_1_ relaxation rate in the presence and absence of 5 mM gadodiamide. Normalized signal intensities plotted against recovery time. (c) ^19^F *R*_1_ values of Trp23 and Trp37 as a function of temperature are plotted as bar graphs, with blue and red indicating Free and Gd^3+^, respectively. Error bars represent fitting errors from exponential curve fitting (b). (d) A plot showing the difference between *R*_1_ (Gd^3+^) and *R*_1_ (free) at each temperature. Larger values indicate greater solvent exposure of the observed ^19^F nuclei. Error bars represent fitting errors from exponential curve fitting (b). (e) Energy landscape of Trp23 flipping in Hsf1 DBD, illustrated based on information from ^19^F NMR approaches.

To evaluate the population shift between the flip‐in and flip‐out states, we monitored temperature‐dependent changes in solvent accessibility using ^19^F sPRE. Because the sPRE effect reflects the population of the flip‐in and flip‐out states, its temperature dependence enables identification of the major and minor states. As a paramagnetic cosolute, we used the neutral and inert agent DTPA‐BMA‐Gd (III) (Gd^3+^), which is well suited for probing solvent accessibility on protein surfaces (Pintacuda & Otting, 2002). We evaluated sPRE based on the longitudinal relaxation rates (*R*_1_) using a saturation recovery experiment (Kitevski‐LeBlanc & Prosser, 2012), both in the presence and absence of 5 mM Gd^3+^. sPRE arises when solvent‐exposed nuclei experience enhanced relaxation (Lenard et al., 2022). Signal intensities were recorded at multiple recovery times and fitted to a single‐exponential function to extract *R*_1_ values for Trp23 and Trp37 (Figures 4a,b and S5). While Trp37 showed negligible change in *R*_1_ upon Gd^3+^ addition, the *R*_1_ of Trp23 increased markedly in the presence of Gd^3+^, indicating significant solvent accessibility (Figure 4c). The population shift between the flip‐in and flip‐out states was evaluated using sPRE by comparing the *R*_1_ with and without Gd^3+^ (*R*_1_ (Gd^3+^) and *R*_1_ (free)) at each temperature. The difference between these values increased more markedly for Trp23 than for Trp37 as the temperature increased (Figure 4c,d). This observation indicates greater solvent exposure of the observed fluorine nucleus in the Trp23 at higher temperatures, suggesting an increased population of the flip‐out state. Accordingly, the flip‐in is identified as the major state and the flip‐out as the minor state. The flip‐out state is expected to induce a void within the protein core, making it structurally less stable, while simultaneously increasing conformational freedom. This expectation was supported by the interpretation that the minor state is enthalpically unfavorable but entropically favorable, consistent with the results of the van't Hoff analysis of the CPMG RD data (Table 1 and Figure S4). Thus, temperature‐dependent sPRE analysis revealed that Trp23 undergoes flipping with the flip‐in and flip‐out states as the major and minor states, respectively.

## 3 DISCUSSION

In this study, we identified a two‐state ring flipping equilibrium of Trp23 in the Hsf1 DBD using a combination of crystallographic data and ^19^F NMR–based approaches. While several crystal structures suggest that Trp23 can adopt either a flip‐in or flip‐out state, our ^19^F NMR data provide evidence that this flipping occurs dynamically in solution. Temperature‐dependent CSP revealed exchange broadening indicative of intermediate exchange, supporting the idea that Trp23 interconverts between these two states on the μs–ms timescale (Figure 1e,f). This interconversion was further quantified by ^19^F CPMG RD, which provided the exchange rate and energy barrier between the two flipping states (Figure 2). Both ^19^F D_2_O isotope shift and sPRE analyses consistently supported the presence of a solvent‐exposed flip‐out state (Figures 3 and 4). In contrast, Trp37, a buried tryptophan residue, exhibited minimal changes under all conditions, validating the specificity and sensitivity of the solvent‐accessibility profiling strategy. Collectively, these results support a structural model in which Trp23 dynamically transitions between a buried and a solvent‐exposed flip state in solution. Furthermore, temperature‐dependent sPRE enabled assignment of the flip‐in/out to the major/minor states. In combination with the parameters obtained from CPMG RD, this allowed for a more detailed visualization of the Trp23 ring flipping in terms of kinetics, energetics, and structural features (Figure 4e).

^19^F NMR is an effective probe for solvent accessibility, particularly when combined with D_2_O isotope shifts and sPRE measurements (Kitevski‐LeBlanc et al., 2009; Zhu et al., 2025). To our knowledge, our work represents the first application of this solvent accessibility profiling to investigate aromatic ring flipping. Owing to its high sensitivity and background‐free detection ^19^F NMR is applicable even to large or membrane proteins (Huang et al., 2020). Although the biological relevance of tryptophan flipping remains largely unexplored, recent studies have shown that ligand binding can modulate the flipping state (Li et al., 2024; Qianzhu et al., 2024), implying potential relevance to molecular recognition. Given the prominent role of tryptophan in stabilizing protein cores (Joel et al., 2014; Minks et al., 1999), probing its dynamics may offer new insights into allosteric regulation and conformational plasticity. Advances in fluorinated amino acid labeling, such as site‐specific incorporation of fluorotryptophan via amber codon suppression systems (Qianzhu et al., 2022), are expanding the scope of ^19^F NMR in protein science. In combination with the ^19^F NMR–based solvent accessibility profiling strategy demonstrated here, these tools provide a powerful approach for visualizing otherwise invisible minor flipping states, thereby enabling broader investigations into the functional roles of aromatic ring dynamics across a range of protein systems.

## 4 MATERIALS AND METHODS

### 4.1 Expression and purification of protein samples

The human Hsf1 DBD (1–120), DBD W23F, and DBD W37F expression constructs were cloned into a pET21b vector (Cat. No. 69741‐3CN; Novagen, Madison, Wisconsin) and fused to GB1‐His^6^ (Weininger et al., 2013) tags at the HRV3C N‐terminus of the protease cleavage site. All constructs were constructed through site‐directed mutagenesis using the PrimeSTAR Mutagenesis Basal Kit (Cat. No. R046A; Takara Bio, Shiga, Japan). All the expression constructs were transformed into BL21(DE3) cells. ^19^F labeled samples were prepared by growing the cells in minimal (M9) medium in the presence of ampicillin (50 μg ml^−1^). 5‐Fluoroindole (Cat. No. F9108; Sigma‐Aldrich, Tokyo, Japan) was added to the culture 0.5 h prior to the addition of IPTG. Subsequently, protein expression was induced by adding 0.5 mM isopropyl‐β‐D‐1‐thiogalactopyranoside (IPTG) at OD_600_ ~ 0.6, followed by a 12 to 16 h incubation at 18°C. Then, the cells were harvested at OD_600_ ~ 3.0, resuspended in a lysis buffer containing 50 mM Tris–HCl (pH 8.0) and 500 mM NaCl, disrupted by a sonicator, and centrifuged at 18,000 rpm for 30 min. The supernatant fraction containing Hsf1 was purified using an Ni‐NTA Sepharose column (Cat. no. 30210; QIAGEN, Hilden, Germany). Additionally, the GB1‐His^6^ tag was removed using a HRV3C protease at 4°C (incubation for 16 h), post which the cleaved Hsf1 DBD was applied onto a Ni‐NTA Sepharose column, and the flow‐through fraction was collected. Samples were further purified by gel filtration using a Superdex 75 pg 16/600 column (Cat. No. 28989333; Cytiva) equilibrated with a solution containing 25 mM MES/NaOH (pH 5.5) and 100 mM NaCl, 0.02% NaN_3_. Finally, protein concentrations were determined spectrophotometrically at 280 nm using the corresponding extinction coefficient.

### 4.2 NMR spectroscopy

NMR samples were prepared in 25 mM MES/NaOH (pH 5.5) and 100 mM NaCl, 0.02% NaN_3_, 10% D_2_O for 1D ^19^F‐NMR experiments. The concentration of Hsf1 DBD was 400 μM. The ^19^F‐NMR experiments were performed at temperatures ranging from 10°C to 30°C. ^19^F‐NMR spectra were obtained with a Bruker AVANCE NEO 800 MHz spectrometer (Bruker, Billerica, MA) using a CPTCI ^1^H/^19^F‐ ^13^C/^15^N proton‐optimized triple resonance cryoprobe. The 1D ^19^F‐NMR experiments were recorded with a data size of 131,072 complex points, an acquisition time of 367 ms, 2048 scans per experiment for temperature‐dependent NMR experiments, 8192 scans per experiment for D_2_O isotope shift, and 1024 scans per experiment for sPRE experiments and CPMG experiments. The spectra were processed using Bruker TOPSPIN version 4.1.4.

### 4.3 ^19^F CPMG RD experiments

The ^19^F CPMG experiments for DBD were recorded with a relaxation delay of 4 ms and with 19 different CPMG frequencies of 0, 250, 500, 750, 1000, 1250, 1500, 1750, 2000, 2250, 2500, 2750, 3000, 3250, 3500, 3750, 4000, 4500, and 5000 Hz at 10, 15, and 20°C. Acoustic ringing artifacts were suppressed using a three‐pulse “aring” sequence (Overbeck et al., 2020). The ^19^F CPMG NMR data were multiplied by an exponential window function with a line broadening factor of 4 Hz prior to Fourier transformation. Two‐site exchange rate constants (*k*_ex_) and the populations (*p*_major_, *p*_minor_ = 1 − *p*_major_) were extracted using GUARDD (Kleckner & Foster, 2012) by fitting the data to the Carver‐Richards‐Jones all‐timescales dispersion equation (Korzhnev et al., 2004).

The activation energy (*E*_a_) for conformational exchange was determined by fitting the temperature dependence of *k*_ex_ values to the Arrhenius equation (1):

(1) $$\ln\left(k_{\mathrm{ex}}\right)=\ln\left(A\right)-E_{a}/\mathrm{RT}$$

where *R* is the universal gas constant (1.987 cal mol^−1^ K^−1^) and *T* is the temperature in Kelvin. The slope was analyzed using Prism 5 (GraphPad Software, San Diego, CA).

The equilibrium constant (*K*_eq_) between the major and minor conformational states was calculated from the population ratio derived from CPMG relaxation dispersion analysis (2):

(2) $$\begin{array}{c} K_{\mathrm{eq}}=p_{\text{minor}}/p_{\text{major}} \end{array}$$

The standard Gibbs free energy difference (Δ*G*°) was then calculated using the following thermodynamic relation (3):

(3) $$\begin{array}{c} \Delta G^{{}^{\circ}}=-\mathrm{RT}\ln\left(K_{\mathrm{eq}}\right) \end{array}$$

To extract enthalpic and entropic contributions to conformational equilibrium, a van't Hoff analysis was performed by plotting ln(*K*_eq_) against 1/*T*, using the van't Hoff equation (4):

(4) $$\begin{array}{c} \ln\left(K_{\mathrm{eq}}\right)=-\Delta H^{{}^{\circ}}/\mathrm{RT}+\Delta S^{{}^{\circ}}/R \end{array}$$

### 4.4 ^19^F D_2_O‐induced isotope shifts

The 100% D_2_O sample of the DBD was prepared by dialyzing a protein solution initially prepared in 25 mM MES/NaOH (pH 5.5), 100 mM NaCl, and 0.02% NaN₃ against 25 mM MES/NaOH (pD 5.5), 100 mM NaCl, 0.02% NaN₃, and 100% D_2_O. The 50% D_2_O sample was prepared by mixing equal volumes of 400 μM DBD solutions prepared in 100% H₂O buffer and 100% D₂O buffer. The 10% D_2_O sample was prepared by adding 10% (v/v) D_2_O directly to a DBD solution in 25 mM MES/NaOH (pH 5.5), 100 mM NaCl, and 0.02% NaN_3_. The ^19^F‐NMR experiments were performed at 20°C.

### 4.5 ^19^F sPRE

^19^F 1D NMR spectra were recorded at recovery delays of 0.15, 0.25, 0.35, 0.50, 1.0, 2.0, 3.0, and 4.0 s in the presence and absence of 5.0 mM DTPA‐BMA‐Gd (III) (Omniscan) (Lenard et al., 2022). For both Trp23‐ and Trp37‐derived signals, peak intensities were normalized such that the intensity at 4.0 s was set to 1. *R*_1_ values were determined by curve fitting using the following equation (5). Fitting errors were used as error bars in the bar graph.

(5) $$\begin{array}{c} I=I_{0}*\left(1-\exp^{\left(-R_{1}t\right)}\right) \end{array}$$

where *I* is the observed signal intensity at time *t*, *I*_0_ is the plateau value (set to 1.0), *R*_1_ is the longitudinal relaxation rate, and *t* is the recovery time (recovery delay + acquisition time + fixed delay).

## AUTHOR CONTRIBUTIONS

**Soichiro Kawagoe:** Conceptualization; data curation; formal analysis; investigation; visualization; writing – original draft; writing – review and editing; funding acquisition; project administration. **Hiroyuki Kumeta:** Investigation; writing – review and editing. **Tomohide Saio:** Conceptualization; writing – review and editing; funding acquisition; project administration; supervision.

## CONFLICT OF INTEREST STATEMENT

The authors declare no conflicts of interest.

## Supporting information


**DATA S1.** Table of χ_1_ − χ_2_ angles of Trp23, electron density maps, sequence alignment, ^19^F NMR spectra with glycerol, van't Hoff plot, ^19^F sPRE of the Hsf1 DBD at each temperature (PDF).

## Data Availability

The data that support the findings of this study are available from the corresponding author upon reasonable request.
