# Supplementary material for: 19F NMR‐based solvent accessibility profiling reveals tryptophan ring‐flip dynamics in a protein
Source: Protein Sci. 2025 Sep 13;34(10):e70307. doi: 10.1002/pro.70307 (PMC12432518; doi:10.1002/pro.70307)
Supplement: Supplementary file 1 — DATA S1. Table of χ1 − χ2 angles of Trp23, electron density maps, sequence alignment, 19F NMR spectra with glycerol, van't Hoff plot, 19F sPRE of the Hsf1 DBD at each temperature (PDF). [file PRO-34-e70307-s001.pdf]

# Supporting Information

# $^{19}$F NMR-based solvent accessibility profiling reveals tryptophan ring-flip dynamics in a protein

Soichiro Kawagoe[1*], Hiroyuki Kumeta[2], and Tomohide Saio[1,3*]

[1] Institute of Advanced Medical Sciences, Tokushima University, Tokushima 770-8503, Japan

[2] Faculty of Advanced Life Science, Hokkaido University, Sapporo, Hokkaido 060-0810, Japan

[3] Fujii Memorial Institute of Medical Sciences, Institute of Advanced Medical Sciences, Tokushima University, Tokushima, 770-8503

[*]Email: kawagoe@tokushima-u.ac.jp, saio@tokushima-u.ac.jp

| PDB ID | $\chi_1$ (deg) | $\chi_2$ (deg) | Flip state | DNA bound state |
|---|---|---|---|---|
| 5d5u | 182.6 | -92.7 | in | Bound |
| 5d5v-B | 196.6 | 82.9 | out | Bound |
| 5d5v-B | 194.4 | 81.6 | out | Bound |
| 5hdg | 194.3 | 93.1 | out | apo |
| 5hdn-A | 202.0 | 93.8 | out | Bound |
| 5hdn-B | 198.3 | 93.6 | out | apo |
| 5hdn-C | 200.8 | 96.0 | out | Bound |
| 5hdn-D | 200.2 | 95.2 | out | apo |
| 7dcj-A | 171.9 | -85.8 | in | Bound |
| 7dcj-B | 174.6 | -84.9 | in | Bound |
| 7dcs-A | 194.3 | 85.7 | out | Bound |
| 7dcs-B | 174.9 | -90.4 | in | Bound |
| 7dcs-C | 179.0 | -91.4 | in | Bound |
| 7dcs-D | 194.7 | 88.4 | out | Bound |
| 7dcs-E | 199.4 | 87.2 | out | Bound |
| 7dcs-F | 171.1 | -91.6 | in | Bound |
| 7dct-A | 197.2 | 88.1 | out | Bound |
| 7dct-B | 167.4 | -88.1 | in | Bound |
| 7dct-C | 180.4 | -90.1 | in | Bound |
| 7dct-D | 193.2 | 90.2 | out | Bound |
| 7dct-E | 180.3 | -89.5 | in | Bound |
| 7dct-F | 204.8 | 88.5 | out | Bound |

**Supplementary Table 1: $\chi_1$-$\chi_2$ angles of Trp23 in Hsf1 DBD, flip state, and DNA-bound state for each PDB structures.**

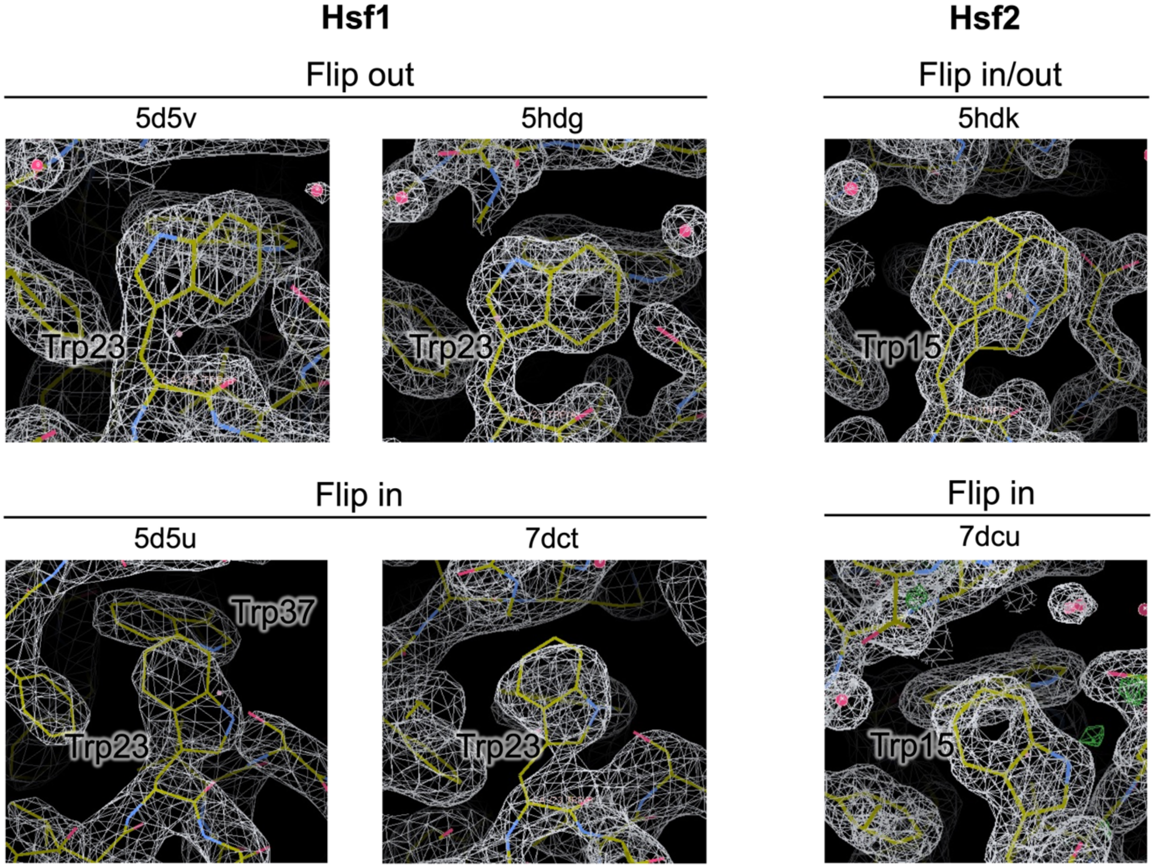


**Figure S1. Electron density around Trp residues in DBD of Hsf1 and Hsf2.** Electron density maps (grey mesh) around the tryptophan residue in crystal structures of Hsf1 (PDB: 5d5v, 5hdg, 5d5u, 7dct) and Hsf2 (PDB: 5hdk, 7dcu). For Trp23 in the Hsf1 DBD and Trp15 in the Hsf2 DBD, crystal structures have been determined for both the flip in and flip out conformations. Maps were visualized in Coot.

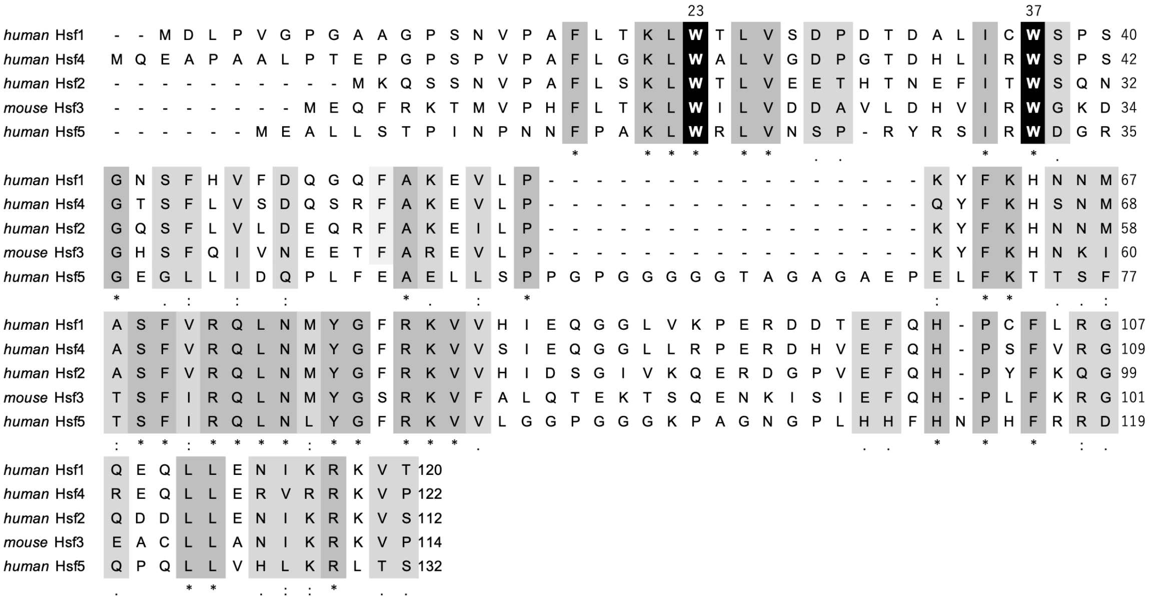


**Figure S2. Conservation of the amino acid residues in the DBD of Hsf family members.** Multiple sequence alignment for DBD of Hsf family, human (Homo sapiens, Q00613) Hsf1, Hsf2, Hsf4, Hsf5, and mouse (Mus musculus, P38532) Hsf3. The sequence for Hsf3 is derived from the mouse ortholog, as a human counterpart is not present. Tryptophan residues are highlighted in black.

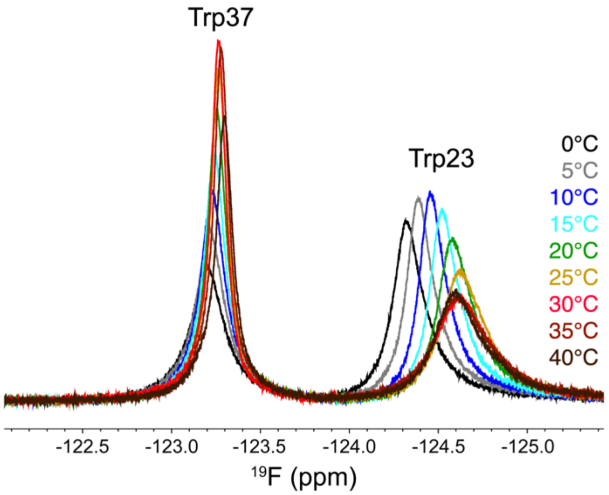


**Figure S3. $^{19}$F NMR spectra of DBD measured from 0°C to 40°C in the presence of 10% glycerol.** Temperature-dependent $^{19}$F NMR spectra of the DBD WT in the presence of 10% glycerol to prevent sample freezing. The Trp23 signal exhibited an upfield shift from 0°C to 10°C, but with increased signal intensity. This likely reflects a reduction in rotational correlation time due to the temperature increase, in addition to contributions from chemical exchange. Between 30°C and 40°C, the Trp23 signal shifted downfield. The signal intensity of Trp37, which had been increasing with temperature, also began to decrease above 30°C, suggesting that partial denaturation or other structural perturbations occur at temperatures above 30°C. Therefore, subsequent experiments and analyses were conducted within the temperature range of 10°C to 30°C in the absence of glycerol.

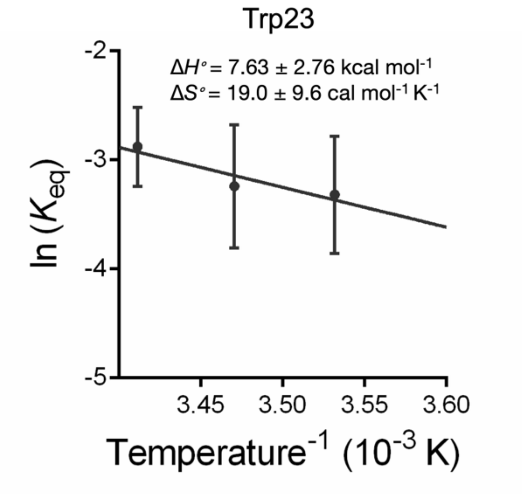


**Figure S4: van't Hoff analysis based on $^{19}$F CPMG experiments.** The equilibrium constants ($K_{eq}$) calculated from the populations of major and minor states obtained from CPMG experiments are plotted against temperature according to the van't Hoff equation.

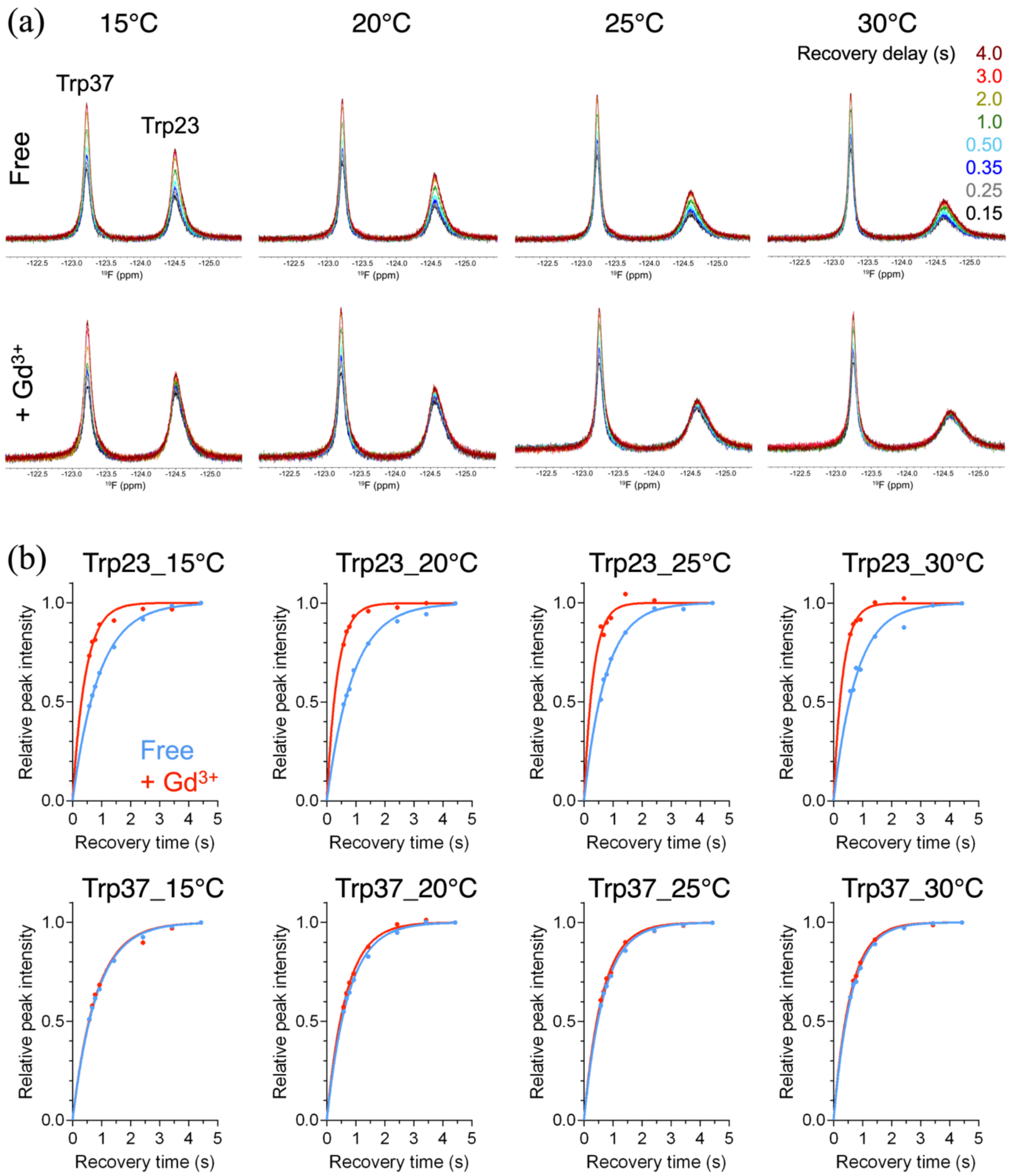


**Figure S5: $^{19}$F solvent PRE of the Hsf1 DBD at each temperature.** (a) Overlay of $^{19}$F NMR spectra of Hsf1 DBD recorded with varying recovery delays ($d_1$ = 0.15 to 4.0 s) in the absence (Free, upper panels) and presence (+$Gd^{3+}$, lower panels) of 5 mM gadodiamide at each temperature. (b) Measurements of $^{19}$F $R_1$ relaxation rate in the presence and absence of 5 mM Gadodiamide. Normalized signal intensities plotted against recovery time.
